# Engagement in a web-based intervention for individuals who committed sexual offenses against children: observational study

**DOI:** 10.1186/s40359-025-02366-z

**Published:** 2025-01-20

**Authors:** Sonja Schröder, Safiye Tozdan, Peer Briken, Jürgen L. Müller, Peter Fromberger

**Affiliations:** 1 University Medical Center Göttingen, Clinic for Psychiatry and Psychotherapy – Forensic Psychiatry, Rosdorfer Weg 70, 37081 Göttingen, Germany; 2 Institute for Sex Research, Sexual Medicine & Forensic Psychiatry, Center for Psychosocial Medicine, University Medical Center Hamburg-Eppendorf, Martinistraße 52, 20246 Hamburg, Germany

**Keywords:** Web-based intervention, Uptake, Engagement, Child sexual abuse, Child sexual exploitation material

## Abstract

Web-based interventions have been shown to be effective for various health and mental health problems. However, the effectiveness of interventions is often limited by the fact that individuals do not start or complete them. Using data from an intermediate analysis of the randomized placebo-controlled clinical trial to evaluate the effectiveness of @myTabu, the current study investigated engagement with a web-based intervention for 113 individuals convicted of child sexual abuse and/or for child sexual exploitation material. @myTabu is a human-supported, web-based intervention for individuals who have been convicted for sexual abuse of children or the use of child sexual exploitation material with the goal of reducing the risk factors for recidivism and actual recidivism. The results of the present study indicate that (a) 81% of the participants logged into the web-based intervention within 8 weeks after login credentials were sent; (b) there was a trend toward lower treatment readiness and lower belief in their own technical prerequisites in individuals who did not login; (c) 15% of the participants completed all requested sessions within the first 5 weeks of participation, and the average participant logged in on 6 different days and completed two sessions; (d) the number of login days and completed sessions could be predicted by treatment readiness. We discuss these findings and their implications for the development and implementation of further web-based interventions in the forensic context. Trial registration: German Clinical Trial Register (DRKS00021256). Prospectively registered 24.04.2020.

**Supplementary Information:**

The online version contains supplementary material available at 10.1186/s40359-025-02366-z.

Web-based interventions (WBIs) are programs in which therapeutic content is presented online in a modularized way through texts, videos, audios, and/or chats. For the most part, users can work through the content themselves but can also receive written feedback through automatic responses and/or humans [1]. With these features, WBIs offer several advantages, as they can be provided flexibly in time and space, anonymously, and cost-effectively [2, 3]. WBIs have shown to be effective in preventing and treating health and mental health problems, such as depression, panic disorder, and posttraumatic stress disorder [4–6]. However, some studies report small (or no) effects of WBIs [7, 8]. These results are commonly explained by the frequently reported lack of engagement with WBI [9–12].

## Engagement in web-based interventions

Many users stop engaging in WBIs for health and mental health problems shortly after they register [13]. This is problematic, as greater engagement is associated with greater effectiveness of a WBI [14]. *Engagement* refers to the behavior of beginning and continuing the use of a WBI [15] and is measured in most studies by examining the amount and depth of engagement behavior [16]. Analytical indicators for the amount of engagement are the rate at which users log into the WBI after registration and the number of further logins [16]. In a meta-analysis on WBI for general mental health, on average, 21% of the randomized participants did not download or use any features of the WBI (*k* = 8, range 0–43%) [17]. An analytical indicator of the depth of engagement is the number of completed sessions. The mean percentage of people who did not complete all sessions in WBIs for general mental health was 36% (*k* = 7, range 8–70%) [17].

The range of engagement can be explained (a) at the study-level for differences between trials and (b) at the participant-level for differences between participants within one trial [17]. At the study-level, the type of WBI has an influence on engagement. In the meta-analysis by Linardon and Fuller-Tyszkiewicz [17], acceptance-based interventions [e.g., Acceptance and Commitment Therapy [18]], sending reminders, offering monetary compensation, and enrollment with contact with a researcher (via telephone or in person) is positively related to engagement [17]. Furthermore, therapeutic or administrative guidance in WBI increases engagement [19].

At the participant-level, there are no conclusive results regarding which characteristics are related to engagement [17]. However, increased engagement is associated with female gender and greater treatment expectancy or credibility [20]. A theoretical framework for studying engagement at the participant-level is provided by the Unified Theory of Acceptance and Use of Technology (UTAUT) [21, 22]. Stemming from information systems research, UTAUT is one of the most studied theoretical models for explaining acceptance and engagement in the context of WBIs [22, 23]. According to UTAUT, engagement (i.e., use behavior) with a technology is determined by the behavioral intention to use it, which is conceptualized as acceptance [24]. Acceptance, in turn, is influenced by performance expectancy, effort expectancy, and social influence. Performance expectancy is defined as the degree to which individuals believe that using a WBI will be helpful. Effort expectancy is defined as the degree of ease in using a WBI. Social influence is defined as the degree to which individuals perceive that people who are important to them would recommend the use of a WBI. In addition to acceptance, facilitating conditions influence engagement. Facilitating conditions are defined as the degree to which individuals believe they have the technical prerequisites to use a WBI and that a technical infrastructure exists to support them. Furthermore, it is assumed that age has a moderating effect on the relationship between facilitating conditions and engagement, indicating that the effect is stronger for older individuals. The UTAUT’s assumption that acceptance can be predicted by performance expectancy, effort expectancy, and social influence has been validated for WBIs for health and mental health problems [23]. Predictors of engagement with a technology have been studied less and moderating variables (age × facilitating conditions) are often not considered [25]. In a meta-analysis of UTAUT in various settings (e.g., health care, education, commerce), Khechine et al. [25] reported that acceptance and facilitating conditions have a positive correlation with a medium effect size with engagement. However, the correlation between facilitating conditions and engagement was very weak and did not pass the failsafe N test [method for controlling a possible publication bias of nonsignificant results [26]] [25]. For acceptance, in the context of WBIs for pain or depression and anxiety, individuals with higher acceptance rates have higher uptake rates and complete more sessions [27, 28].

### Web-based interventions for individuals who committed sexual offenses against children

In addition to the application of WBIs for health and mental health, WBIs are also being developed in the forensic setting to prevent recidivism in individuals who have committed child sexual abuse (CSA) offenses or child sexual exploitation material (CSEM) offenses [3, 29]. A systematic review on the use of technology in the treatment for individuals who have committed a crime revealed that advantages for WBIs for health and mental health problems are also relevant in the forensic setting: time and costs could be saved, physical barriers such as traveling distance, or access to treatment in highly secured settings could be overcome, and the threshold to participate in treatment could be reduced because of enhanced privacy and anonymity [30].

To date, studies on the effectiveness of WBIs for individuals who committed CSA or CSEM offenses are scarce [29]. In 2022, Lätth et al. [31] evaluated a human-supported WBI, called Prevent It, for individuals who commit CSEM offenses. The participants were recruited mainly via Darknet forums (i.e., chat forums and links regarding pedophilia or CSEM on the Darknet) and could take part anonymously. The results showed that, compared with users of a psychological placebo WBI (*n* = 78), users of the WBI Prevent It (*n* = 76) reported a statistically significant, albeit small decrease in weekly viewing of CSEM [31]. This (small) effect size might be related to the engagement of the participants. 17% of the 160 participants did not complete any session (uptake, defined as at least one login to the WBI, was not reported) and 74% of the participants failed to complete all the sessions. On average the participants completed 4.31 (*SD* = 2.75) out of 8 sessions [31].

### Engagement in web-based interventions for individuals who committed sexual offenses against children

There are no studies on the factors influencing engagement with WBIs for individuals who committed CSA and/or CSEM offenses [30]. The results from research on face-to-face (f2f) treatment for individuals who committed offenses are often contradictory at the participant-level. Factors such as age, level of education, or type of sexual offence that were correlated with engagement in one study had no effect in other studies [32, 33]. This is presumably due to differences between the treatment type and population studied or the operationalizing of engagement during the trials [34]. However, two concepts that are often associated with treatment engagement are risk and responsivity [32, 34]. Risk and responsivity are two concepts from the RNR (risk-need-responsivity) model, which conceptualizes effective correctional programs for individuals who committed an offense [35]. According to the concept of “risk”, treatment intensity should be adjusted to the individual’s likelihood of re-offending. According to the concept of “responsivity”, adjusting the treatment to the individual’s learning style, personality, and cognitive capacities by using behavioral, social learning, and cognitive behavioral strategies, such as cognitive behavioral therapy, is important [35]. A concept that builds on the concept of responsivity but also accounts for the interrelation of different processes and structures within responsivity is treatment readiness [36]. Treatment readiness describes the characteristics of the patient or the therapeutic situation that are suitable for promoting engagement [32]. High levels of recidivism risk and low treatment readiness could both lead to low levels of engagement [32].

### Objective

The goals of the present study were to determine engagement (uptake, login days, and session completion) in the WBI @myTabu in a sample of individuals who committed CSA and/or CSEM offenses and to investigate predictors of engagement during the randomized, placebo-controlled clinical trial to evaluate the effectiveness of @myTabu [37]. In addition to the core predictors of engagement from the UTAUT model (i.e., acceptance and facilitating conditions), the additional predictors recidivism risk and treatment readiness were included in the model. Furthermore, we tested whether age moderates the relationship between facilitating conditions and engagement. In summary, the following research questions were addressed:

1. What are the (a) uptake, (b) login days, and (c) session completion rates in a WBI for individuals who committed CSA and/or CSEM offenses?
2. Do acceptance, facilitating conditions, recidivism risk, and treatment readiness predict (a) uptake, (b) login days, and (c) session completion? What are the moderating effects of age on the relationship between facilitating conditions and engagement?

We thus tested the hypotheses that higher scores in acceptance, facilitating conditions, and treatment readiness and lower scores in recidivism risk are related to higher uptake probability, more login days and more completed sessions.

## Method

### Study design and data collection

In the present observational study, the data from all participants of the first recruitment year (March 1, 2021, to March 1, 2022) of the clinical WBI @myTabu effectiveness trial [37, 38] are included. This interim analysis was conducted so that a data safety monitoring board could confirm the study’s safety, allowing it to continue without increasing the risk of re-offending. Furthermore, the data were used for additional evaluations, such as in the study by Schröder et al. [39] on acceptance of WBIs, in which the same sample was analyzed as in the present study. To avoid influencing the final analyses, the interim data were not unblinded.

For the recruitment of participants, scientific staff informed community supervisors about the clinical trial and asked them to inform eligible individuals under their supervision. Eligibility criteria in the clinical trial are as follows: (a) conviction of CSA and/or use of CSEM under the German Criminal Code; (b) community supervision period of at least 6 months; (c) adulthood (18 years of age or older); (d) internet access; and (e) no severe acute psychiatric disorder (e.g., severe depression or severe schizophrenia), no severe cerebro-organic disorder, and no severe cognitive impairment. The decision as to whether the inclusion criteria were met was based on the statements of the community supervisor and on the statements of the interested individuals at a personal interview with a scientific staff member whereby interested, eligible individuals were provided with information about the study. Data were obtained through a personal interview and coding of the court file, which was conducted after written informed consent was obtained from the participants. The participants were instructed to complete the Questionnaire for Acceptance of Technology (ATQ) [37, 39], a pen and paper questionnaire that assessed acceptance and its predictors, as well as the Corrections Victoria Treatment Readiness Questionnaire (CVTRQ) [40]. The court file consisting of the written court judgment and records of the Federal Central Criminal Register was coded by a scientific staff member.

Once enrolled in the study, community supervisors received participant login credentials and were asked to share them with participants at their next meeting or sooner (e.g., by postally). With the login credentials, participants could log in to the @myTabu website via an internet-enabled device. On the website, participants had to complete the introductory session and, after that, weekly sessions. After completing the introductory session and the first content session, the next session was unlocked 7 days after the start of the previous session [37]. The participants were credited with monetary compensation after completing a session, which they could request as a voucher if they had saved €15 (or a multiple thereof). The total amount of compensation was €120 when all WBI sessions were completed (1 introductory session and 24 content sessions).

### Web-based intervention

In the study, participants were randomly assigned to either the experimental WBI (i.e., @myTabu) or a placebo WBI. Both WBIs start with the same introductory session, in which features of the WBI are explained. The WBIs further consist of six thematic modules with four content sessions each. In the experimental WBI, the topics of the modules are motivation, supervision and social relationships, emotion management, problem solving, offense-supportive attitudes, and sexuality. In the placebo WBI, the topics of the modules are physical activities, sleep, dreams, nutrition, and well-being. The content sessions consisted of psychoeducational material (e.g., texts, videos, audio files, images) and exercises (e.g., fill-in-the-gap, open text, multiple choice quiz) which were displayed one after the other in the sessions. New psychoeducational material or exercise only opens when the previous one has been clicked as read or the exercise has been edited. For the exercises, participants receive automated feedback during the session or feedback from an online coach after the session is completed. A session is considered to be completed when all psychoeducational material and all exercises have been completed in the described manner. For more details on the WBI, see Fromberger et al. [37] and, for German readers, Bauer et al. [38].

### Measures

#### Sociodemographic and criminological data

The sociodemographic and criminological data were derived from written court judgments, records of the Federal Central Criminal Register and interviews with the participants. Information on the additional data that were coded during that process can be found in the study protocol of the @myTabu clinical trial [37].

#### Acceptance and facilitating conditions

To measure acceptance and facilitating conditions, the Questionnaire for Acceptance of Technology (ATQ) was developed within the @myTabu project [37]. The ATQ is based on the German adaptation of the UTAUT questionnaire items developed by Baumeister et al. [41] and Apolinário-Hagen et al. [42]. The items are derived from the widely established UTAUT questionnaire [22] and from adaptations of this questionnaire to the field of health and mental health [43–46]. For the purposes of the present study, the items were modified to be suitable for a WBI in the forensic setting, with modifications based on considerations of face validity.

Acceptance was operationalized as the behavioral intention to use a WBI and was evaluated via four items: (a) “I can imagine trying an online program”, (b) “I can imagine using an online program regularly”, (c) “I would recommend an online program to a friend”, and (d) “I would be willing to pay for an online program”. Facilitating conditions were operationalized as the belief in technical infrastructure and technical support when using a WBI and were evaluated via two items: (a) “I do have all necessary technical preconditions for using an online program” and (b) “In the case of technical problems with an online program, I would receive technical support”. The participants were asked to respond on a 5-point Likert scale, with the range of responses ranging from 1 (*does not apply at all*) to 5 (*applies absolutely*). The items of the scales were averaged and had possible scores ranging from 1 to 5 with higher scores representing greater acceptance and a stronger belief in facilitating conditions. The McDonald ω total for acceptance was 0.59 [47]. For facilitating conditions, the Spearman‒Brown-coefficient was 0.33 [48]. The reliability of acceptance can be considered poor, and that of facilitating conditions can be considered unacceptable [49].

In addition to acceptance and facilitating conditions, the ATQ measures performance expectancy (helpfulness of the WBI), effort expectancy (ease of use of a WBI), social influence (social environments’ opinions relating to a WBI), attitude toward WBIs (overall affective reaction to using a WBI), internet anxiety (fear of making mistakes on the internet), planning behavior (planning when the WBI is to be used), and importance of study compensation (monetary compensation and awards in the WBI). The scales were answered on the same 5-point Likert scale, except for planning behavior, which was adopted from Zarski et al. [50] and was answered on a 4-point Likert scale ranging from 1 (*not true at all*) to 4 (*exactly true*). The items of the scales were averaged and had possible scores ranging from 1 to 5, besides planning behavior, which had a possible range of scores from 1 to 4. Higher scores represent a stronger belief in the WBI’s helpfulness, easy use, recommendation of the social environment, a more positive attitude toward the WBI, a greater fear of the internet, a more detailed plan of when to work on the WBI, and stronger importance of study compensation. The McDonald ω total was 0.80 for performance expectancy, 0.81 for effort expectancy, 0.83 for attitude toward WBIs, and 0.88 for planning behavior [47]. The Spearman‒Brown-coefficients for the scales with two items were 0.21 for social influence, 0.65 for internet anxiety, and 0.63 for importance of study compensation [48]. The reliability of performance expectancy, effort expectancy, attitude toward WBIs, and planning behavior can be considered good. For social influence, reliability can be considered unacceptable, and for importance of study compensation and internet anxiety the reliability can be considered questionable [49]. These scales were included only in the explorative analyses. A description of all items and modifications is provided in Supplementary Table 1.

#### Treatment readiness

Treatment readiness was measured with the Corrections Victoria Treatment Readiness Questionnaire (CVTRQ), which measures the readiness to change and to engage in the treatment of individuals who committed a crime [40]. The questionnaire consists of 20 items and includes the following scales: (a) attitudes and motivation, (b) emotional reaction, (c) offending beliefs, and (d) efficacy. The items are rated on a 5-point Likert scale, with the range of responses ranging from 1 (*strongly disagree*) to 5 (*strongly agree*). Treatment readiness is composed of the averaged sum of the scales and has a possible range of scores from 1 to 5. Higher scores represent greater treatment readiness.

For the explorative analysis, the subscales of the CVTRQ were averaged and had possible scores ranging from 1 to 5. Higher scores represent a more positive view toward treatment and motivation to change, more negative emotional responses to one’s own criminal behavior, stronger sense of responsibility to one’s own criminal behavior, and greater confidence in one’s own abilities and in others. It was shown in the development of the CVTRQ that the questionnaire correlates with similar measures, such as the Readiness to Change Questionnaire [40, 51]. In addition, studies on individuals who committed offenses and are undergoing f2f treatment have shown that the CVTRQ or scales of the questionnaire can differentiate between individuals who have not completed or started f2f treatment and individuals who have completed the treatment [52–54].

Currently, to our knowledge, no German translation of the CVTRQ exists. Therefore, a German version was created via translation and back-translation within the @myTabu-consortium [37]. The McDonald ω total for the total score was 0.78 [47] which can be considered as acceptable [49]. McDonald ω total for attitudes and motivation was 0.63, for emotional reaction 0.56, for offending beliefs 0.67, and for efficacy 0.50 [47], which can be considered poor for emotional reaction and efficacy and questionable for attitudes and motivation and offending beliefs [49].

#### Recidivism risk

The present study assesses the risk of recidivism by utilizing a modified version of the actuarial assessment instrument Static-99 [55] translated into German [56]. For the modification, the approach of Seto et al. [57] was followed, in which the victim-related items 8, 9, and 10 were removed from the scoring. This was done to create a uniform risk score for individuals convicted of CSA or CSEM. The modified Static-99 can take integer values between zero and nine. A higher value represents a greater risk of recidivism. The Static-99 score was determined on the basis of written judgment, records of the Federal Central Criminal Register, and information obtained from the participants.

#### Uptake, login days, and session completion

Uptake, login days, and session completion were measured by analyzing the log-file data of the @myTabu website. Uptake was operationalized dichotomously and was coded as “yes” if the participant logged into the WBI within eight weeks after the login credentials were sent to the community supervisor. The period of eight weeks was chosen to give participants sufficient time to receive the login credentials and to log in to the WBI.

The login days were operationalized by counting the days on which logins into the WBIs took place within 5 weeks after the first login day. Session completion was operationalized by counting the completed sessions within 5 weeks after the first login. The 5-week period for login days and completion of sessions was chosen because participants signed an intervention contract at enrollment in which they committed to completion of one session per week. Within the first 5 weeks, they should have been able to complete the introductory session and the four content sessions of the module motivation [38]. Owing to technical restrictions, a maximum of six sessions could be completed within the first 5 weeks of the study. However, one participant was able to complete seven sessions during this time. Since this additional session was solely due to a technical malfunction, the number of completed sessions was altered to six.

### Sample characteristics

The present study included data from *N* = 113 male participants with a median age of 38 years (*M* = 40.67, *SD* = 12.75), with an age range of 20–72 years. On average, they had 1.25 previous convictions of any type (*SD* = 2.47), with 56.6% (64/113, with one missing value) first-time convictions. Of the 48 individuals who had previous convictions, 21 individuals had solely conducted sexual offenses in the past, 15 individuals had solely committed other types of offenses, and 12 individuals had conducted sexual offenses and other types of offenses. In terms of the index offense, 38.9% (44/113) of the participants were convicted of CSA [§ 176 [58], in the version in effect before July 01, 2021], 14.2% (16/113) of aggravated CSA [§ 176a [58], in the version in effect before July 01, 2021], and 74.3% (84/113) of dissemination, procurement, and possession of CSEM [§ 184b [58]]. Note that 28 participants had more than one present conviction as index offense, so the percentages do not add to 100%. The mean score of the modified Static-99 was 1.87 (*SD* = 1.19, range 0–6). Table 1 lists descriptive statistics of the scales investigated in the ATQ and the CVTRQ.

**Table 1:** Descriptive statistics of the ATQ scales and the CVTRQ total score and subscales

Variables	Md	M	SD
ATQ scales			
Acceptance	*4.00*	*3.78*	*0.66*
Facilitating conditions	*4.50*	*4.41*	*0.68*
Technical prerequisites	*5.00*	*4.72*	*0.59*
Source of support	*4.00*	*4.11*	*1.02*
Performance expectancy	*4.00*	*4.08*	*0.77*
Effort expectancy	*4.00*	*4.10*	*0.67*
Social influence	*4.00*	*3.88*	*0.81*
Close people	*3.00*	*3.24*	*1.20*
Supervisor	*5.00*	*4.48*	*0.80*
Attitude	*4.25*	*4.15*	*0.63*
Internet anxiety	*2.00*	*2.02*	*0.93*
Planning behavior	*3.00*	*2.91*	*0.82*
Study compensation	*2.50*	*2.60*	*1.05*
CVTRQ total score			
Treatment readiness	*4.20*	*4.18*	*0.40*
CVTRQ subscales			
Attitudes and motivation	*4.33*	*4.31*	*0.51*
Emotional reaction	*4.17*	*4.15*	*0.58*
Offending beliefs	*4.50*	*4.41*	*0.57*
Efficacy	*3.75*	*3.82*	*0.54*

Note *N* = 113. All scales have a possible range of scores ranging from 1 to 5, besides planning behavior, which has a possible range of scores from 1 to 4. ATQ = Questionnaire for Acceptance of Technology. CVTRQ = Corrections Victoria Treatment Readiness Questionnaire. Technical prerequisites = Having the technical prerequisites. Source of support = Existing technical infrastructure to help them. Close people = Recommendation by close people. Supervisor = Recommendation by community supervisor. Attitude = Attitude toward web-based interventions

### Statistical analyses

Data analysis was performed using the software R, version 4.2.1 [59]. The authors take responsibility for the integrity of the data, the accuracy of the data analyses, and have made every effort to avoid inflating statistically significant results by preregistering the analysis (AsPredicted number 107092) and by correcting for alpha inflation.

For the first research question (see Objectives), measures of center and variance for uptake, login days, and the number of completed sessions were calculated, and their distributions were assessed with histograms. For the second research question, multiple regression models were used. In accordance with the preregistration, participants with a response rate of less than 70% on all questionnaire scales analyzed in the present study would have been excluded from the sample [60]. On the basis of this criterion, no participants were excluded. For instances of missing item-level data for the scales acceptance, facilitating conditions, and treatment readiness, the scale-wise mean across available items was calculated. Seven participants exhibited missing items, with a maximum of five missing items (*M* = 2.4). All participants provided at least one response per scale, thus, no scale-wise missing data were observed in the present study. The preregistration specified exclusion criteria on the basis of cutoff dates so that participants whose login credentials were sent after January 03, 2022, and those who logged in after January 24, 2022, were not included. However, during the interim analysis, we obtained data beyond these dates. Consequently, we revised the criteria to include participants on the basis of a consistent cutoff of 8 weeks for uptake (*N* = 113) and 5 weeks for login days and session completion (*N* = 99). This allowed for a larger sample size.

To analyze the criterion uptake, we planned to perform a logistic regression in the preregistration. However, because of the small sample size in the group that did not log in once, the rule of ten was not met [61]. Therefore, we exploratively investigated whether there was a difference in the predictors between those who logged into the intervention and those who did not by performing Mann‒Whitney U tests with Holm’s alpha correction [62]. Mann‒Whitney U tests were performed as the distribution of the scales significantly deviated from normality according to the Shapiro‒Wilk test [63]. Because of the low Spearman‒Brown-coefficient of facilitating conditions, the two items of the scale were looked at separately.

To analyze the criteria login days and session completion, a Poisson regression with the predictor variables acceptance, facilitating conditions, treatment readiness, recidivism risk, and the moderator age on the effects of facilitating conditions (age × facilitating conditions) was calculated. A significance level of 0.05 was adopted. Predictors were included simultaneously in the model and were mean-centered before calculation to allow meaningful interpretation of the coefficients of the first-order terms in the presence of interactions [64]. For the outcomes login days and session completion, the overdispersion test [65] was significant (*z* = 4.31, *p* < .001; *z* = 3.01, *p* = .001, respectively), which supported the use of a negative binomial model. The Vuong test [66] did not support the use of zero-inflated models over standard negative binomial models, *z* = − 1.55, *p* = .06; *z* = − 1.52, *p* = .06, respectively. Therefore, negative binomial models were applied. With the likelihood-ratio test, the models were compared with the constant-only models. As the models did not provide significant improvement in predicting login days and session completion compared to the constant-only models, a stepwise backward and forward selection procedure was used exploratively to build the most parsimonious prediction models. The models conducted via this procedure were then compared with the constant-only models. The incidence rate ratio was obtained by exponentiating the regression coefficient. The incidence rate ratios represent the multiplicative change in the expected count for each one-unit increase in the predictor variable.

According to the preregistration, the associations of all scales of the ATQ (performance expectancy, effort expectancy, social influence, attitude toward WBIs, internet anxiety, planning behavior, importance of study compensation) with the outcome uptake, login days, and session completion was looked at in explorative analyses. To analyze uptake, Mann‒Whitney U tests with Holm’s alpha correction were conducted. Mann‒Whitney U tests were performed as the distribution of the scales significantly deviated from normality according to the Shapiro‒Wilk test [63]. To analyze login days, a negative binomial model was applied (overdispersion test: *z* = 5.06, *p* < .001; Vuong test: *z* = 3.01, *p* = .001). To analyze session completion, a zero-inflated, negative binomial model was applied that combines a negative binomial count model with the preregistered predictors and a constant-only logistic zero model (overdispersion test: *z* = 3.42, *p* < .001; Vuong test: *z* = − 2.60, *p* = .005).

In addition to the preregistered explorative analyses, the associations of all subscales of the CVTRQ (attitudes and motivation, emotional reaction, offending beliefs, efficacy) with the outcome uptake, login days, and session completion were analyzed. To analyze uptake, Mann‒Whitney U tests with Holm’s alpha correction were conducted. Mann‒Whitney U tests were performed as the distribution of the scales significantly deviated from normality according to the Shapiro‒Wilk test [63]. To analyze login days and session completion, zero-inflated, negative binomial models were applied that combined a negative binomial count model with the subscales as predictors and a constant-only logistic zero model (overdispersion test: *z* = 4.81, *p* < .001; Vuong test: *z* = − 3.01, *p* = .001; overdispersion test: *z* = 3.05, *p* = .001; Vuong test: *z* = − 1.93, *p* = .03, respectively).

For the calculations, various R-packages were used: the Mann‒Whitney U test was conducted using the coin package, version 1.4.2 [67]; the likelihood-ratio test was conducted using the lmtest package, version 0.9.40 [68]; the overdispersion test was conducted using the AER package, version 1.2.10 [69]; the Vuong test was conducted using the pscl package, version 1.5.5.1 [70]; the negative binomial model and the stepwise backward and forward selection procedure were conducted using the MASS package, version 7.3.57 [71]; and the zero-inflated, negative binomial model was calculated using the glmmTMB package, version 1.1.7 [72].

## Results

### Research question 1: uptake, login days, and session completion

Within 8 weeks after their login credentials were sent to the community supervisor, 80.5% (91/113) of the 113 participants logged into the WBI. The median time for the first login was 2.60 weeks (*M* = 3.08, *SD* = 1.76, range 0.43–7.30). Among the 22 participants who did not log in during the first 8 weeks, 40.9% (9/22) logged in at a later time. Their time for the first login ranged from 8.07 to 66.63 weeks.

Within the first 5 weeks after the first login day, the median number of days of access to the WBI was 5 days (*M* = 5.99, *SD* = 5.73, range 0–27 days), and the median number of logins was 6 (*M* = 9.71, *SD* = 12.21, range 0–67). Among the participants, 15.2% (15/99) did not access the WBI on a further day after the day of their first login. Within these 5 weeks, the median number of completed sessions was two (*M* = 2.31, *SD* = 1.93, range 0–6 sessions). Among the participants, 21.2% (21/99) did not complete any session, and 21.2% (21/99) completed the introductory session only. The introductory session and at least the first four content sessions were completed by 15.2% (15/99) of the participants.

### Research question 2: prediction of uptake, login days, and session completion

#### Uptake

Mann‒Whitney U tests showed a trend toward lower treatment readiness for individuals who did not log in than for those people who logged in, *z* = − 2.63, *p* = .051. There was no difference between individuals who did not log into the WBI and individuals who logged into the WBI for acceptance, facilitating conditions, or risk. Analysis of the items of the facilitating conditions scale revealed a trend that individuals who did not log in had lower ratings of their technical prerequisites for using an online program than did individuals who logged in, *z* = − 2.55, *p* = .053. There was no trend in the rating of how they perceived the source of support in the case of technical problems (see Table 2).

**Table 2:** Differences between individuals who did not log into the WBI and individuals who logged into the WBI

Predictor	Md		M (SD)		z-value	*r*	*p*
	No ^a^	Yes ^b^	No ^a^	Yes ^b^			
Acceptance	3.5	4.0	3.6 (0.6)	3.8 (0.7)	–1.90	0.18	0.23
Facilitating conditions	4.5	4.5	4.1 (0.9)	4.5 (0.6)	–1.80	0.17	0.23
Technical prerequisites	5.0	5.0	4.4 (0.8)	4.8 (0.5)	–2.55	0.24	0.053
Source of support	4.0	4.0	3.9 (1.2)	4.2 (1.0)	-1.19	0.11	0.47
Treatment readiness	4.0	4.3	4.0 (0.5)	4.2 (0.4)	–2.63	0.25	0.051
Risk	2.0	2.0	2.0 (1.5)	1.8 (1.1)	0.03	0.003	0.97

Note Mann‒Whitney U Tests with Holm’s Alpha Correction. No = Individuals who did not log into the WBI within 8 weeks. Yes = Individuals who logged into the WBI within 8 weeks. Technical prerequisites = Having the technical prerequisites. Source of support = Existing technical infrastructure to help them

^a^*n* = 22. ^b^*n* = 91

#### Login days

Table 3 shows the results of the negative binomial regression with login days as the outcome. Higher treatment readiness predicted a greater number of login days. The model did not predict login days better than the constant-only model did, χ² [5] = 9.72, *p* = .08. The stepwise backward and forward selection procedure showed that the most parsimonious model is the one with the predictor treatment readiness. The model performed better in predicting login days than the constant-only model did, χ² [1] = 7.19, *p* = .007. Additionally, in this model, higher treatment readiness predicted a greater number of login days with a one-unit increase in treatment readiness increasing the login days by a factor 2.10 while holding all other variables in the model constant (see Table 3).

**Table 3:** Negative binomial regression before and after the stepwise selection procedure for the prediction of login days

Predictor	B (raw)	SE	z-value	*p*	e^B^ (incident rate ratios)
Before stepwise selection procedure					
(Intercept)	1.74	0.10	17.45	< 0.001	
Acceptance	0.15	0.18	0.88	0.38	1.17
Facilitating conditions	–0.16	0.17	–0.97	0.33	0.85
Treatment readiness	0.74	0.35	2.14	0.03	2.10
Risk	0.03	0.11	0.31	0.76	1.03
Facilitating conditions × Age	–0.01	0.01	–0.53	0.59	0.99
After stepwise selection procedure					
(Intercept)	1.75	0.10	17.37	< 0.001	
Treatment readiness	0.74	0.28	2.66	0.008	2.10

*Note N* = 99

#### Session completion

Table 4 shows the results of the negative binomial regression with session completion as the outcome. The model did not predict session completion better than the constant-only model did, χ² [5] = 11.02, *p* = .051. The stepwise backward and forward selection procedure showed that the most parsimonious model is the one with the predictor treatment readiness. The model performed better predicting session completion than the constant-only model did, χ² [1] = 8.64, *p* = .003. Higher treatment readiness predicted a greater number of completed sessions with a one-unit increase in treatment readiness increases the number of completed sessions by a factor 2.05 while holding all other variables in the model constant (see Table 4).

**Table 4:** Negative binomial regression after the stepwise selection procedure for the prediction of session completion

Predictor	B (raw)	SE	z-value	*p*	e^B^ (incident rate ratios)	
Before stepwise selection procedure						
(Intercept)	0.80	0.08	9.52	< 0.001		
Acceptance	0.15	0.15	1.03	0.31	1.17	
Facilitating conditions	–0.08	0.14	–0.59	0.55	0.92	
Treatment readiness	0.68	0.30	2.30	0.02	1.98	
Risk	0.03	0.09	0.39	0.70	1.03	
Facilitating conditions × Age	0.01	0.01	0.97	0.33	1.01	
After stepwise selection procedure						
(Intercept)	0.81	0.09	9.46	< 0.001		
Treatment readiness	0.72	0.24	2.94	0.003	2.05	

Note *N* = 99

### Explorative analysis

Mann‒Whitney U tests showed a trend toward lower social influence for individuals who did not log in than for individuals who logged in, *z* = − 2.62, *p* = .08. Looking at the items of the scale of social influence separately, the difference between the groups is stronger in the item querying the belief that people close to the participant would recommend participation in a WBI, with lower scores for individuals who did not log in than for those who logged in. The other variables showed no significant differences or trends (see Table 5).

**Table 5:** Differences in the ATQ scales between individuals who did not log in and individuals who logged into the WBI

Predictor	Md		M (SD)		z-value	*r*	*p*
	No ^a^	Yes ^b^	No ^a^	Yes ^b^			
Performance expectancy	3.7	4.3	3.7 (0.9)	4.2 (0.7)	–2.31	0.22	0.16
Effort expectancy	4.0	4.3	4.0 (0.7)	4.1 (0.7)	–0.61	0.06	0.99
Social influence	3.5	4.0	3.5 (0.9)	4.0 (0.8)	–2.62	0.25	0.08
Close people	3.0	3.0	2.7 (1.2)	3.4 (1.2)	–2.22	0.21	0.19
Supervisor	4.5	5.0	4.2 (1.1)	4.6 (0.7)	–1.44	0.14	0.89
Attitude	4.0	4.3	4.0 (0.7)	4.2 (0.6)	–1.39	0.13	0.89
Internet anxiety	2.0	2.0	2.1 (1.2)	2.0 (0.9)	–0.09	0.01	0.99
Planning behavior	3.0	3.0	2.8 (0.9)	2.9 (0.8)	–0.63	0.06	0.99
Study compensation	2.8	2.5	2.8 (1.0)	2.6 (1.1)	1.00	0.09	0.99

Note Mann‒Whitney U Tests with Holm’s Alpha Correction. ATQ = Questionnaire for Acceptance of Technology. No = Individuals who did not log into the WBI within 8 weeks. Yes = Individuals who logged into the WBI within 8 weeks. Close people = Recommendation by close people. Supervisor = Recommendation by community supervisor. Attitude = Attitude toward web-based interventions

^a^*n* = 22. ^b^*n* = 91

A negative binomial regression for predicting login days and a zero-inflated, negative binomial regression for predicting session completion did not predict the respective outcomes better than the constant-only model did, χ² [9] = 16.17, *p* = .06 and χ² [9] = 6.37, *p* = .70. None of the predictors were significant (see Tables 6 and 7).

**Table 6:** Negative binomial regression for the association between login days and the ATQ scales

Predictor	B (raw)	SE	z-value	*p*	e^B^ (incident rate ratios)
(Intercept)	–1.15	1.03	–1.12	0.26	
Acceptance	0.19	0.18	1.06	0.29	1.21
Facilitating conditions	–0.05	0.17	–0.28	0.78	0.95
Performance expectancy	0.27	0.17	1.57	0.12	1.31
Effort expectancy	–0.01	0.18	–0.05	0.96	0.99
Social influence	–0.12	0.16	–0.75	0.45	0.89
Attitude	0.16	0.21	0.76	0.45	1.18
Internet anxiety	0.19	0.12	1.57	0.12	1.21
Planning behavior	0.18	0.15	1.14	0.26	1.19
Study compensation	0.06	0.09	0.63	0.53	1.06

Note *N* = 99. ATQ = Questionnaire for Acceptance of Technology. Attitude = Attitude toward web-based interventions

**Table 7:** Zero-inflated, negative binomial regression for the association between session completion and the ATQ scales

Predictor	B (raw)	SE	z-value	*p*	e^B^ (incident rate ratios)
Counts portion of the model					
(Intercept)	–0.66	1.05	–0.63	0.53	
Acceptance	0.18	0.16	1.14	0.26	1.19
Facilitating conditions	–0.01	0.14	–0.04	0.97	0.99
Performance expectancy	0.08	0.14	0.54	0.59	1.08
Effort expectancy	0.06	0.14	0.42	0.67	1.06
Social influence	0.01	0.14	0.04	0.97	1.01
Attitude	0.14	0.19	0.71	0.48	1.14
Internet anxiety	0.05	0.10	0.54	0.59	1.06
Planning behavior	–0.16	0.13	–1.22	0.39	0.85
Study compensation	0.04	0.08	0.54	0.59	1.05
Logistic portion of the model					
(Intercept)	–2.22	0.93	–2.40	0.02	

Note *N* = 99. ATQ = Questionnaire for Acceptance of Technology. Attitude = Attitude toward web-based interventions

Mann‒Whitney U tests showed a trend toward lower attitudes and motivation (*z* = − 2.41, *p* = .06), emotional reaction (*z* = − 2.28, *p* = .07), and offending beliefs (*z* = − 2.20, *p* = .07) for individuals who did not log in than for individuals who logged in. The subscale efficacy showed no significant difference or trend (see Table 8).

**Table 8:** Differences in the CVTRQ subscales between individuals who did not log into and individuals who logged into the WBI

Predictor	Md		M (SD)		z-value	*r*	*p*
	No ^a^	Yes ^b^	No ^a^	Yes ^b^			
Attitudes and motivation	4.0	4.3	4.1 (0.5)	4.4 (0.5)	–2.41	0.23	0.06
Emotional reaction	3.9	4.2	3.9 (0.7)	4.2 (0.5)	–2.28	0.21	0.07
Offending beliefs	4.3	4.8	4.1 (0.8)	4.5 (0.5)	–2.20	0.21	0.07
Efficacy	3.8	3.8	3.8 (0.6)	3.8 (0.5)	–0.25	0.02	0.81

Note Mann‒Whitney U Tests with Holm’s Alpha Correction. CVTRQ = Corrections Victoria Treatment Readiness Questionnaire. No = Individuals who did not log into the WBI within 8 weeks. Yes = Individuals who logged into the WBI within 8 weeks

^a^*n* = 22. ^b^*n* = 91

The zero-inflated, negative binomial regression performed better in predicting login days than the constant-only model did, χ² [4] = 10.34, *p* = .04. A greater emotional reaction predicted a greater number of login days with a one-unit increase in emotional reaction increasing the login days by a factor 1.65 while holding all other variables in the model constant (see Table 9).

**Table 9:** Zero-inflated, negative binomial regression for the association between login days and the CVTRQ subscales

Predictor	B (raw)	SE	z-value	*p*	e^B^ (incident rate ratios)
Counts portion of the model					
(Intercept)	–1.21	1.04	–1.16	0.25	
Attitudes and motivation	0.13	0.22	0.61	0.54	1.14
Emotional reaction	0.50	0.20	2.46	0.01	1.65
Offending beliefs	–0.02	0.24	–0.07	0.95	0.98
Efficacy	0.11	0.19	0.59	0.56	1.12
Logistic portion of the model					
(Intercept)	–2.43	0.64	–3.76	< 0.001	

Note *N* = 99. CVTRQ = Corrections Victoria Treatment Readiness Questionnaire

The zero-inflated, negative binomial regression model performed better in predicting session completion than the constant-only model did, χ² [4] = 10.62, *p* = .03. Higher attitudes and motivation predicted a greater number of completed sessions with a one-unit increase in attitudes and motivation increasing the number of completed sessions by a factor 1.45 while holding all other variables in the model constant (see Table 10).

**Table 10:** Zero-inflated, negative binomial regression for the association between session completion and the CVTRQ subscales

Predictor	B (raw)	SE	z-value	*p*	e^B^ (incident rate ratios)
Counts portion of the model					
(Intercept)	–1.68	1.01	–1.66	0.10	
Attitudes and motivation	0.37	0.19	1.98	0.048	1.45
Emotional reaction	0.26	0.15	1.67	0.10	1.29
Offending beliefs	0.04	0.20	0.19	0.85	1.04
Efficacy	–0.07	0.15	–0.48	0.63	0.93
Logistic portion of the model					
(Intercept)	–2.02	0.53	–3.84	< 0.001	

Note *N* = 99. CVTRQ = Corrections Victoria Treatment Readiness Questionnaire

## Discussion

This is the first study that investigates the extent to which individuals who committed CSA and/or CSEM offenses and who are under community supervision engage with a WBI. The analysis revealed that a high proportion of participants (81%) logged into the WBI within 8 weeks after the login credentials were sent to the respective community supervisor. Of the participants who logged in at least once, 85% logged into WBI on another day, but only 15% completed all the sessions they were asked to complete within the first 5 weeks. An explorative analysis revealed a trend that treatment readiness was lower in the group of individuals who did not log in. A further explorative analysis of the two items comprising the facilitating conditions scale revealed that there was a trend that the belief in their own technical prerequisites was greater in individuals who logged into the WBI. The same is, however, not true of their belief in receiving support when facing technical problems. Login days and session completion were predicted by treatment readiness. Participants with higher treatment readiness were more likely to login on more days and to complete more sessions. In contrast to the initial hypothesis, the predictors acceptance, facilitating conditions, and recidivism risk predicted neither login days nor session completion. Furthermore, age was not a moderator on the relationship between facilitating conditions and treatment.

In an explorative analysis of all scales measured with the ATQ, we see a trend that social influence was greater in individuals who logged into the WBI. This trend was stronger for believing that people close to the participant would recommend participation in a WBI and weaker for believing that the community supervisors would recommend participation. Furthermore, none of the scales predicted login days or session completion. The explorative analysis of the subscales of the CVTRQ showed a trend towards attitudes and motivation, emotional reaction, and offending beliefs being greater in individuals who logged into the WBI. Further, login days were predicted by emotional reaction and session completion was predicted by attitudes and motivation.

### Engagement in a WBI

Judging the relative success of the present WBI to engage participants is difficult because of a lack of similar WBIs. The WBI which is most comparable to that of the present study, seems to be *Prevent It* [31]. In a clinical trial, Lätth et al. [31] observed that of the 160 anonymous participants, 83% completed at least one session, and 26% completed all the sessions. Despite differences in study design (identified vs. anonymous participants) and participant characteristics (e.g., reported vs. unreported crime), the uptake rate in the present study (81%) is similar to that for completion of at least one session (83%) in the study by Lätth et al. [31]. These values are comparable with the mean percentage of 79% of people using a WBI targeting general mental health after registration [17]. The similar uptake in both studies is unexpected, given that previous research has demonstrated higher attrition rates in trials where participants can enroll without personal contact with a researcher than in trials that require such contact for enrollment [17]. A possible explanation for this is that the recruitment method used in the present study might also have included individuals who are less technically skilled, which was not an obstacle during their personal enrollment in the study. However, during online registration, they might have opted out because of their (perceived) lower technical proficiency. This hypothesis contrasts with the finding that almost all participants rated their own technical prerequisites as high in the present study. Nevertheless, a ceiling effect of the ratings in the present study and that the actual technical prerequisites of the participants were higher in the study by Lätth et al. [31] cannot be ruled out.

In the present study, 15% of the participants who logged into the WBI successfully completed all the sessions within the requested timeframe of 5 weeks. This is less than the 26% completion rate for the whole WBI reported by Lätth et al. [31]. In both studies, the participants were asked to complete one session per week. However, in the study by Lätth et al. [31], participation was terminated if the time limit of 1 week was exceeded; in the present study, participants could also continue the WBI at a later date. The difference in completion rates might indicate that, when given more time constraints, more people complete the WBI in the predefined time. This concurs with the results of the qualitative study by Gerhards et al. [73], in which participants appreciated the ability to engage with the WBI at their own time and pace. However, they also stated that “someone or something forcing you” helped to adhere to the WBI. In the present study, community supervisors were informed when participants did not log in within a month and were asked to remind their clients of their participation. The degree to which community supervisors actually do this and the degree to which they motivate their clients could vary greatly among different community supervisors. In addition to the time constraints, the WBIs and participants were very different in the two studies thus the delivery method might be only one of many factors influencing the different completion rates. However, in a study by Viskovich and Pakenham, the delivery method of the WBI found to be unrelated to the completion rate [74].

### Predictors of engagement

When examining the two groups of individuals who logged into the WBIs and those who did not log in during the first 8 weeks after login credentials were sent, we observed a trend that treatment readiness was greater in individuals who logged into the WBI, with a small to medium effect size. A further explorative analysis revealed a trend that this effect could be attributed to a more positive view of treatment and motivation to change, a more negative emotional responses to one’s own criminal behavior, and a stronger sense of responsibility for one’s own criminal behavior in individuals who logged into the WBI. Furthermore, treatment readiness was the only significant predictor of login days and session completion. In an explorative analysis, the emotional reaction subscale was a significant predictor of login days, and the attitudes and motivation subscale was a significant predictor of session completion. The concept of treatment readiness comes from f2f treatment. F2f treatment studies showed that individuals who did not start [52] or did not complete [53, 54] a treatment could be differentiated by subscales of treatment readiness. In those previous studies in the f2f context the subscales attitudes and motivation [53, 54] and efficacy were particularly relevant for engagement [52]. The present study indicates that the concept stemming from f2f treatment might also be transferable to the context of WBIs. The explorative analysis indicates that the subscales attitudes and motivation, emotional reaction, and offending beliefs might be most relevant. In the WBI @myTabu, the first module (comprising four sessions), which started after the introductory session addresses the responsivity factor *lack of motivation* and uses Motivational Interviewing [75] to strengthen the participants’ motivation to use the WBI and to change their behavior [37, 38, 76]. However, those individuals who logged into the WBI and worked on those sessions were those who had greater treatment readiness and presumably greater motivation to change and were thus the individuals who might not have this responsivity factor. Therefore, it might be useful to include engagement-facilitation interventions addressing treatment readiness and motivation to change at study enrollment — especially for those who show a lower treatment readiness. This could be done, for example, by using Motivational Interviewing [75]. It may be most fruitful to focus on the advantages of treatment and behavioral change (attitudes and motivation), the disadvantages of one’s own criminal behavior (emotional reaction), and enhancing the sense of responsibility to one’s own criminal behavior (offending beliefs). Furthermore, specific “if–then” plans (e.g., “If I do not feel like completing a session, then I review my treatment goals”) could be implemented, which have been shown to increase the continued use of WBIs [50].

In addition, even though almost all participants rated their own technical prerequisites as high, there was a trend that the belief in their own technical preconditions was lower among individuals who had logged into the WBIs than among individuals who had not logged in. Again, this may indicate that personal enrollment promoted access to the WBI for individuals who might not have registered if they had had to register online. Furthermore, this highlights the importance of participants being technically well-prepared, for example, having the necessary electronic devices and/or knowing how to operate them. Previous studies have shown that engagement-facilitation interventions, including training and education on the use of WBIs (e.g., by providing assistance during first registration), increase uptake rates [77]. Thus, to increase uptake rates, it appears to be important to ensure the technical ability of the participant to actually log into the WBI.

In the exploratory analyses, there was a trend that social influence, especially the belief that people close to the participant would recommend participation, was greater in those individuals who logged into the WBI. Individuals who perceive strong recommendations by people close to them might also receive more help or motivation in their personal environment, which could lead to a higher probability of logging into a WBI. No predictor included in the exploratory analysis significantly predicted login frequency or session completion. The lack of significant results in these exploratory analyses might mean that there were aspects in addition to psychological predictors that influenced participation. These could be personal predictors (e.g., available time [20], or intellectual and reading abilities [78]), characteristics of the participant’s presenting situation [20] (e.g., open court proceedings), or intervention- and computer-related predictors (e.g., experience with WBIs or the internet [20, 73], confusion between research questionnaires and the WBI [73], or active administrative support [20, 77], e.g., by the community supervisor).

### Limitations

The first limitation is related to use of data from an intermediate analysis. Therefore, it was not possible to analyze differences in engagement between the intervention group and the placebo group in the clinical trial. Studies show that membership in the intervention- or control-group can influence engagement; however, the direction of the effect varies across studies [20]. For the present study, the process leading up to the first login and the introductory session are the same for both groups. The intervention and placebo groups start to differ after this first introductory session, when the content sessions begin. Accordingly, uptake cannot be influenced by group membership. However, it may be that group membership had an impact on login days and session completion.

The second limitation is that the ATQ scales acceptance, facilitating conditions, and social influence and the CVTRQ subscales emotional reaction and efficacy show low reliability. The ATQ used in the present study is based on the well-established UTAUT questionnaire [22] with adaptations to the field of health and mental health [43–46]. The questions were altered only slightly to fit the field of forensic psychiatry (see Supplementary Table 1). For the CVTRQ, the original questionnaire was translated into German. The low reliability could be a sign that parts of the questionnaire may not be directly transferable to the context of the present study [39], for example, because the participants interpreted the items differently than in studies on WBIs for health and mental health. For facilitating conditions, low reliability is in line with previous results, in which facilitating conditions were the scale with the lowest reliability [41] or were not calculated, as it was not considered to be a uniform scale [27, 43]. For all (sub)scales with low reliability, this may indicate that the concepts of the scales were not measured correctly, therefore, the scales may not be valid. A modification of the items or an increase in the number of items could be considered for further studies to improve the internal consistency of the measured constructs. When doing this, it should be ensured that the scales measure the underlying concept, for example, by comparing the scales with other questionnaires that measure a similar concept. Within the present study, the low internal consistency was taken into account to the extent that for scales with two items, these items were considered individually in the analyses in addition to the entire scale.

The third limitation is related to the use of the modified Static-99 to assess the risk of recidivism [57]. To date, there are no published studies that show the predictive accuracy of the (modified) Static-99 score for the recidivism of this target group [79]. There are, however, studies that suggest that other established risk assessment tools for individuals who committed CSA offenses, such as the Risk Matrix 2000, may also be predictive of recidivism in individuals who commit CSEM offenses [79, 80]. Furthermore, Eke et al. [81] reported that a modified Static-99R score predicts any sexual, contact sexual, and CSEM recidivism, at least when used in a sample of individuals convicted for CSEM or CSEM and CSA. When the sample was restricted to individuals who were solely convicted for CSEM offenses, Static-99R did not predict recidivism. However, the observed samples were small, and the recidivism rates were low. Use of the (modified) Static-99 to predict recidivism for individuals who are solely convicted for CSEM offenses is thus not justifiable [82] and would require a study of the relationship between the (modified) Static-99 and recidivism with a greater sample of individuals who committed CSEM offenses [81].

## Conclusion

This study showed that 81% of participants logged into the intervention within 8 weeks. This rate is comparable to the average uptake rate in WBIs for general mental health. Furthermore, treatment readiness (likely especially attitudes and motivation, emotional reaction, and offending beliefs), technical prerequisites, and supportive surroundings might be less common in individuals who committed CSA and/or CSEM offenses and who did not log in. Thus, engagement-facilitation interventions addressing treatment readiness, technical prerequisites, and the social surroundings of the user might help to enhance the uptake of WBIs in this specific context. After the first login, the majority of participants did not complete all the sessions they were asked to complete within the first 5 weeks. To enhance long-term use, treatment readiness appears to be important, and within treatment readiness, attitudes and motivation, as well as emotional reaction could be especially relevant. These insights contribute to the development and implementation of effective WBIs for individuals who committed CSA and/or CSEM offenses.

## Electronic supplementary material

Below is the link to the electronic supplementary material.

Supplementary Material 1

## Data Availability

The English translation of the ATQ is included as Supplementary Table 1. All datasets generated and/or analyzed during the current study are not publicly available. Parts of the data are highly sensitive (e.g., conviction of sexual abuse of children) and cannot be fully anonymized. Parts of the dataset can be made available upon reasonable request from the corresponding author.

## Abbreviations

ATQ: Questionnaire for Acceptance of Technology
CSA: Child sexual abuse
CSEM: Child sexual exploitation material
CVTRQ: Corrections Victoria Treatment Readiness Questionnaire
f2f: Face-to-face
RNR: Risk-need-responsivity
UTAUT: Unified Theory of Acceptance and Use of Technology
WBI: Web-based intervention

## References

[1] Barak A, Klein B, Proudfoot JG. Defining internet-supported therapeutic interventions. Ann Behav Med. 2009;38(1):4–17. 19787305 10.1007/s12160-009-9130-7

[2] Hedman E, Furmark T, Carlbring P, Ljótsson B, Rück C, Lindefors N et al. A 5-Year follow-up of internet-based cognitive behavior therapy for social anxiety disorder. J Med Internet Res 2011; 13(2). 10.2196/jmir.1776 PMC3221374 21676694

[3] Wild TSN, Fromberger P, Jordan K, Müller I, Müller JL. Web-based health services in forensic psychiatry: a review of the use of the internet in the treatment of child sexual abusers and child sexual exploitation material offenders. Front Psychiatry 2019; 9. 10.3389/fpsyt.2018.00763 PMC6369176 30778306

[4] Hedman E, Ljótsson B, Lindefors N. Cognitive behavior therapy via the internet: a systematic review of applications, clinical efficacy and cost-effectiveness. Expert Rev Pharmacoecon Outcomes Res. 2012;12(6):745–64. 23252357 10.1586/erp.12.67

[5] Karyotaki E, Ebert DD, Donkin L, Riper H, Twisk J, Burger S, et al. Do guided internet-based interventions result in clinically relevant changes for patients with depression? An individual participant data meta-analysis. Clin Psychol Rev. 2018;63:80–92. 29940401 10.1016/j.cpr.2018.06.007

[6] Hedman-Lagerlöf E, Carlbring P, Svärdman F, Riper H, Cuijpers P, Andersson G. Therapist-supported internet-based cognitive behaviour therapy yields similar effects as face-to-face therapy for psychiatric and somatic disorders: an updated systematic review and meta-analysis. World Psychiatry. 2023;22(2):305–14. 37159350 10.1002/wps.21088 PMC10168168

[7] Kooij L, Groen WG, van Harten WH. The effectiveness of information technology-supported shared care for patients with chronic disease: a systematic review. J Med Internet Res 2017; 19(6). 10.2196/jmir.7405 PMC5500776 28642218

[8] Hadjiconstantinou M, Byrne J, Bodicoat DH, Robertson N, Eborall H, Khunti K et al. Do web-based interventions improve well-being in type 2 diabetes? A systematic review and meta-analysis. J Med Internet Res 2016; 18(10). 10.2196/jmir.5991 PMC5097175 27769955

[9] Kelders SM, Kok RN, Ossebaard HC, van Gemert-Pijnen LJEWC. Persuasive system design does matter: a systematic review of adherence to web-based interventions. J Med Internet Res 2012; 14(6). 10.2196/jmir.2104 PMC3510730 23151820

[10] Eysenbach G. The law of attrition. J Med Internet Res 2005; 7(1). 10.2196/jmir.7.1.e11 PMC1550631 15829473

[11] Chiu TML, Eysenbach G. Stages of use: consideration, initiation, utilization, and outcomes of an internet-mediated intervention. BMC Med Inf Decis Mak 2010; 10. 10.1186/1472-6947-10-73 PMC3000372 21092275

[12] Sin J, Galeazzi G, McGregor E, Collom J, Taylor A, Barrett B et al. Digital interventions for screening and treating common mental disorders or symptoms of common mental illness in adults: systematic review and meta-analysis. J Med Internet Res 2020; 22(9). 10.2196/20581 PMC7495259 32876577

[13] Lattie EG, Schueller SM, Sargent E, Stiles-Shields C, Tomasino KN, Corden ME, et al. Uptake and usage of IntelliCare: a publicly available suite of mental health and well-being apps. Internet Interv. 2016;4(2):152–8. 27398319 10.1016/j.invent.2016.06.003 PMC4936531

[14] Gan DZQ, McGillivray L, Han J, Christensen H, Torok M. Effect of engagement with digital interventions on mental health outcomes: a systematic review and meta-analysis. Front Digit Health. 2021;3:764079. 34806079 10.3389/fdgth.2021.764079 PMC8599127

[15] Cavanagh K. Turn on, tune in and [don’t] drop out: engagement, adherence, attrition, and alliance with internet-based interventions. In: Bennett-Levy J, Farrand P, Christensen H, Griffiths KM, Kavanagh D, Klein B, et al. editors. Oxford guide to low intensity CBT interventions. New York: Oxford University Press; 2010. pp. 227–33.

[16] Pham Q, Graham G, Carrion C, Morita PP, Seto E, Stinson JN et al. A library of analytic indicators to evaluate effective engagement with consumer mHealth apps for chronic conditions: scoping review. JMIR Mhealth Uhealth 2019; 7(1). 10.2196/11941 PMC6356188 30664463

[17] Linardon J, Fuller-Tyszkiewicz M. Attrition and adherence in smartphone-delivered interventions for mental health problems: a systematic and meta-analytic review. J Consult Clin Psychol. 2020;88(1):1–13. 31697093 10.1037/ccp0000459

[18] Hayes SC, Strosahl KD, Wilson KG. Acceptance and Commitment Therapy: second addition, the process and practice of mindful change. New York: Guilford Press; 2011.

[19] Richards D, Richardson T. Computer-based psychological treatments for depression: a systematic review and meta-analysis. Clin Psychol Rev. 2012;32(4):329–42. 22466510 10.1016/j.cpr.2012.02.004

[20] Beatty L, Binnion C. A systematic review of predictors of, and reasons for, adherence to online psychological interventions. Int J Behav Med. 2016;23(6):776–94. 26957109 10.1007/s12529-016-9556-9

[21] Ryan C, Bergin M, Wells JSG. Theoretical perspectives of adherence to web-based interventions: a scoping review. Int J Behav Med. 2018;25(1):17–29. 28730402 10.1007/s12529-017-9678-8

[22] Venkatesh V, Morris MG, Davis GB, Davis FD. User acceptance of information technology: toward a unified view. MIS Q. 2003;27(3):425–78.

[23] Philippi P, Baumeister H, Apolinário-Hagen J, Ebert DD, Hennemann S, Kott L et al. Acceptance towards digital health interventions—model validation and further development of the Unified Theory of Acceptance and Use of Technology. Internet Interv 2021; 26. 10.1016/j.invent.2021.100459 PMC8463857 34603973

[24] Holden RJ, Karsh B-T. The technology acceptance model: its past and its future in health care. J Biomed Inf. 2010;43(1):159–72. 10.1016/j.jbi.2009.07.002 PMC2814963 19615467

[25] Khechine H, Lakhal S, Ndjambou P. A meta-analysis of the UTAUT model: Eleven years later. Can J Adm Sci. 2016;33(2):138–52.

[26] Rosenthal R. The file drawer problem and tolerance for null results. Psychol Bull. 1979;86(3):638–41.

[27] Lin J, Faust B, Ebert DD, Krämer L, Baumeister H. A web-based acceptance-facilitating intervention for identifying patients’ acceptance, uptake, and adherence of internet- and mobile-based pain interventions: Randomized controlled trial. J Med Internet Res 2018; 20(8). 10.2196/jmir.9925 PMC6123541 30131313

[28] Gulliver A, Calear AL, Sunderland M, Kay-Lambkin F, Farrer LM, Batterham PJ. Predictors of acceptability and engagement in a self-guided online program for depression and anxiety. Internet Interv 2021; 25. 10.1016/j.invent.2021.100400 PMC8122006 34026569

[29] Schröder S, Bauer L, Müller JL, Briken P, Fromberger P, Tozdan S. Web-based interventions for individuals who committed sexual offenses against children: development, evaluation, and implementation. Criminal Justice Behav. 2023;50(2):235–51.

[30] Kip H, Bouman YHA, Kelders SM, van Gemert-Pijnen LJEWC. eHealth in treatment of offenders in forensic mental health: a review of the current state. Front Psychiatry 2018; 9. 10.3389/fpsyt.2018.00042 PMC5826338 29515468

[31] Lätth J, Landgren V, McMahan A, Sparre C, Eriksson J, Malki K et al. Effects of internet-delivered cognitive behavioral therapy on use of child sexual abuse material: a randomized placebo-controlled trial on the Darknet. Internet Interv 2022; 30. 10.1016/j.invent.2022.100590 PMC9789379 36573073

[32] Larochelle S, Diguer L, Laverdière O, Greenman PS. Predictors of psychological treatment noncompletion among sexual offenders. Clin Psychol Rev. 2011;31(4):554–62. 21239099 10.1016/j.cpr.2010.12.004

[33] Olver ME, Wong S. Predictors of sex offender treatment dropout: psychopathy, sex offender risk, and responsivity implications. Psychol Crime Law. 2011;17(5):457–71.

[34] Brunner F, Neumann I, Yoon D, Rettenberger M, Stück E, Briken P. Determinants of dropout from correctional offender treatment. Front Psychiatry. 2019;10:142. 30967799 10.3389/fpsyt.2019.00142 PMC6440434

[35] Bonta J, Andrews DA. Risk–need–responsivity model for offender assessment and rehabilitation. Ottawa: Public Safety Canada; 2007 [cited 2024 Feb 21]. Available from: URL: https://www.securitepublique.gc.ca/cnt/rsrcs/pblctns/rsk-nd-rspnsvty/rsk-nd-rspnsvty-eng.pdf

[36] Ward T, Day A, Howells K, Birgden A. The multifactor offender readiness model. Aggress Violent Beh. 2004;9(6):645–73.

[37] Fromberger P, Schröder S, Bauer L, Siegel B, Tozdan S, Briken P et al. @myTabu—A placebo controlled randomized trial of a guided web-based intervention for individuals who sexually abused children and individuals who consumed child sexual exploitation material: a clinical study protocol. Front Psychiatry 2021; 11. 10.3389/fpsyt.2020.575464 PMC7820175 33488416

[38] Bauer L, Schröder S, Tozdan S, Müller JL, Fromberger P. @myTabu—Konzept Einer Therapeuten-gestützten online-intervention für verurteilte Personen, die Kindesmissbrauch Begangen Oder Missbrauchsabbildungen konsumiert haben. Bewährungshilfe. 2021;68(1):5–23.

[39] Schröder S, Buntrock C, Neumann L, Müller JL, Fromberger P. Acceptance of a web-based intervention in individuals who committed sexual offenses against children: cross-sectional study. JMIR Form Res 2024; 8. 10.2196/48880 PMC10858427 38277200

[40] Casey S, Day A, Howells K, Ward T. Assessing suitability for offender rehabilitation: development and validation of the treatment readiness questionnaire. Criminal Justice Behav. 2007;34(11):1427–40.

[41] Baumeister H, Nowoczin L, Lin J, Seifferth H, Seufert J, Laubner K, et al. Impact of an acceptance facilitating intervention on diabetes patients’ acceptance of internet-based interventions for depression: a randomized controlled trial. Diabetes Res Clin Pract. 2014;105(1):30–9. 24862240 10.1016/j.diabres.2014.04.031

[42] Apolinário-Hagen J, Hennemann S, Fritsche L, Drüge M, Breil B. Determinant factors of public acceptance of stress management apps: Survey study. JMIR Ment Health 2019; 6(11). 10.2196/15373 PMC6873149 31697243

[43] Baumeister H, Seifferth H, Lin J, Nowoczin L, Lüking M, Ebert DD. Impact of an acceptance facilitating intervention on patients’ acceptance of internet-based pain interventions: a randomized controlled trial. Clin J Pain. 2015;31(6):528–35. 24866854 10.1097/AJP.0000000000000118

[44] Kim J, Park H-A. Development of a health information technology acceptance model using consumers’ health behavior intention. J Med Internet Res 2012; 14(5). 10.2196/jmir.2143 PMC3510715 23026508

[45] Or CKL, Karsh B-T, Severtson DJ, Burke LJ, Brown RL, Brennan PF. Factors affecting home care patients’ acceptance of a web-based interactive self-management technology. J Am Med Inf Assoc. 2011;18(1):51–9. 10.1136/jamia.2010.007336 PMC3005875 21131605

[46] Wilson EV, Lankton NK. Modeling patients’ acceptance of provider-delivered e-health. J Am Med Inf Assoc. 2004;11(4):241–8. 10.1197/jamia.M1475 PMC436070 15064290

[47] Hayes AF, Coutts JJ. Use omega rather than Cronbach’s alpha for estimating reliability. But… Commun Methods Meas. 2020;14(1):1–24.

[48] Eisinga R, te Grotenhuis M, Pelzer B. The reliability of a two-item scale: Pearson, Cronbach, or Spearman-Brown? Int J Public Health. 2013;58(4):637–42. 23089674 10.1007/s00038-012-0416-3

[49] George D, Mallery P. IBM SPSS statistics 26 step by step: a simple guide and reference. 16th ed. New York: Routledge; 2019.

[50] Zarski A-C, Berking M, Reis D, Lehr D, Buntrock C, Schwarzer R et al. Turning good intentions into actions by using the health action process Approach to predict adherence to internet-based depression prevention: secondary analysis of a randomized controlled trial. J Med Internet Res 2018; 20(1). 10.2196/jmir.8814 PMC5785685 29326097

[51] Rollnick S, Heather N, Gold R, Hall W. Development of a short ‘readiness to change’ questionnaire for use in brief, opportunistic interventions among excessive drinkers. Br J Addict. 1992;87(5):743–54. 1591525 10.1111/j.1360-0443.1992.tb02720.x

[52] Hatcher RM, Roberts ALI. Can completers, non-completers, and non-starters of community-based offending behavior programs be differentiated by internal treatment readiness factors? Int J Offender Ther Comp Criminol. 2019;63(7):1066–81. 30526177 10.1177/0306624X18813891

[53] Shaw J, Edelmann R. Personality disorder, treatment readiness and dropout from treatment in three community-based cognitive skills and violence reduction programmes. Jnl Forensic Pract. 2017;19(4):247–57.

[54] Bosma AQ, Kunst MJJ, Dirkzwager AJE, Nieuwbeerta P. Treatment readiness as a determinant of treatment participation in a prison-based rehabilitation program: an exploratory study. Int J Offender Ther Comp Criminol. 2017;61(8):857–73. 26399465 10.1177/0306624X15605609

[55] Harris A, Phenix A, Hanson RK, Thornton D. Static-99: coding rules revised—2003. Ottawa: Solicitor General Canada; 2003.

[56] Rettenberger M, Eher R. Actuarial assessment of sex offender recidivism risk: a validation of the German version of the Static-99. Sex Offender Treat. 2006;1(3):1–11.

[57] Seto MC, Wood JM, Babchishin KM, Flynn S. Online solicitation offenders are different from child pornography offenders and lower risk contact sexual offenders. Law Hum Behav. 2012;36(4):320–30. 22849417 10.1037/h0093925

[58] German Criminal Code, Strafgesetzbuch SGB. 2021 [cited 2023 Aug 17]. Available from: URL: https://www.gesetze-im-internet.de/englisch_stgb/

[59] R Core Team. R: A language and environment for statistical computing. R Foundation for statistical Computing, Vienna, Austria. 2022. Available from: URL: https://www.R-project.org/

[60] Newman DA. Missing data: five practical guidelines. Organ Res Methods. 2014;17(4):372–411.

[61] Peduzzi P, Concato J, Kemper E, Holford TR, Feinstein AR. A simulation study of the number of events per variable in logistic regression analysis. J Clin Epidemiol. 1996;49(12):1373–9. 8970487 10.1016/s0895-4356(96)00236-3

[62] Holm S. A simple sequentially rejective multiple test procedure. Scand J Stat. 1979;6(2):65–70.

[63] Shapiro SS, Wilk MB. An analysis of variance test for normality (complete samples). Biometrika. 1965;52(3–4):591–611.

[64] Aguinis H, Edwards JR, Bradley KJ. Improving our understanding of moderation and mediation in strategic management research. Organ Res Methods. 2017;20(4):665–85.

[65] Cameron A, Trivedi PK. Regression-based tests for overdispersion in the Poisson model. J Econ. 1990;46(3):347–64.

[66] Vuong QH. Likelihood ratio tests for model selection and non-nested hypotheses. Econometrica. 1989;57(2):307–33.

[67] Hothorn T, Hornik K, van de Wiel MA, Zeileis A. A lego system for conditional inference. Am Stat. 2006;60(3):257–63.

[68] Zeileis A, Hothorn T. Diagnostic checking in regression relationships. R News. 2002; 2(3):7–10. Available from: URL: https://CRAN.R-project.org/doc/Rnews/

[69] Kleiber C. Applied econometrics with R. New York: Springer; 2008. (Use R).

[70] Jackman S, pscl. Classes and methods for R. Developed in the Political Science Computational Laboratory, Stanford University. Department of Political Science, Stanford University, Stanford, CA. R package version 1.03.5; 2010. Available from: URL: https://cir.nii.ac.jp/crid/1572261549901049728

[71] Venables WN, Ripley B. Modern applied statistics with S-PLUS. Fourth Edition. New York: Springer; 2002.

[72] Brooks ME, Kristensen K, van Benthem KJ, Magnusson A, Berg CW, Nielsen A, et al. glmmTMB balances speed and flexibility among packages for zero-inflated generalized linear mixed modeling. R J. 2017;9(2):378.

[73] Gerhards SAH, Abma TA, Arntz A, de Graaf LE, Evers SMAA, Huibers MJH, et al. Improving adherence and effectiveness of computerised cognitive behavioural therapy without support for depression: a qualitative study on patient experiences. J Affect Disord. 2011;129(1–3):117–25. 20889214 10.1016/j.jad.2010.09.012

[74] Viskovich S, Pakenham KI. Pilot evaluation of a web-based acceptance and commitment therapy program to promote mental health skills in university students. J Clin Psychol. 2018;74(12):2047–69. 29962090 10.1002/jclp.22656

[75] Miller WR, Rollnick S. Motivational interviewing: preparing people for change. 2nd ed. New York: Guilford Press; 2002.

[76] Beyko MJ, Wong SCP. Predictors of treatment attrition as indicators for program improvement not offender shortcomings: a study of sex offender treatment attrition. Sex Abuse. 2005;17(4):375–89. 16341600 10.1177/107906320501700403

[77] Leach B, Parkinson S, Gkousis E, Abel G, Atherton H, Campbell J et al. Digital facilitation to support patient access to web-based primary care services: scoping literature review. J Med Internet Res 2022; 24(7). 10.2196/33911 PMC9335178 35834301

[78] Callahan PA, Jeglic EL, Calkins C. Sexual offenders with intellectual disabilities: an exploratory comparison study in an incarcerated U.S. sample. Int J Offender Ther Comp Criminol. 2022;0(0):1–22. 10.1177/0306624X211066825 34963349

[79] Helmus LM, Eke AW, Farmus L, Seto MC. The CPORT and Risk Matrix 2000 for men convicted of child sexual exploitation material (CSEM) offenses: a predictive accuracy comparison and meta-analysis. Criminal Justice Behav. 2024;51(1):3–23.

[80] Wakeling HC, Howard P, Barnett G. Comparing the validity of the RM2000 scales and OGRS3 for predicting recidivism by internet sexual offenders. Sex Abuse. 2011;23(1):146–68. 21349831 10.1177/1079063210375974

[81] Eke AW, Babchishin KM, Paquette S, Scott SB, Batinic M, Fortin F, et al. Comparing the Static-99R and the child Pornography Offender Risk Tool (CPORT) in two Canadian samples adjudicated of child sexual exploitation material offenses. J Criminal Justice. 2024;95:102303.

[82] Helmus LM, Eke AW, Seto MC. What risk assessment tools can be used with men convicted of child sexual exploitation material (. CSEM) offenses? Recommendations from a review of current research; 2024. 10.1037/lhb0000594 39946588

