# Supplementary material for: Engagement in a web-based intervention for individuals who committed sexual offenses against children: observational study
Source: BMC Psychol. 2025 Jan 20;13:59. doi: 10.1186/s40359-025-02366-z (PMC11748531; doi:10.1186/s40359-025-02366-z)
Supplement: Supplementary file 1 — Supplementary Material 1 [file 40359_2025_2366_MOESM1_ESM.docx]

# Supplementary Material

**Supplementary Table 1**

*Items of the Questionnaire on Acceptance and Facilitating Conditions with References to Original Studies*

| Questionnaire Items |
| --- |
| **Acceptance**   1. I can imagine trying an *online program*. ^a^ 2. I can imagine using an *online program* regularly *[…]*. ^a^ 3. I would recommend an *online program* to a friend. ^a^ 4. I would be willing to pay for an *online program*. ^a^   **Performance Expectancy**   1. Using an *online program* would *help me not to commit a further child abuse or to consume child sexual exploitation material*. ^a^ 2. Using an *online program* would *improve my ability to live a crime-free life*. ^a^ 3. Overall, an *online program* would help me *during my community supervision*. ^a^   **Effort Expectancy**   1. Using an *online program* would be simple. ^a^ 2. Using an *online program* would be an easy task for me. ^a^ 3. An *online program* would be clear and easily comprehensible to me. ^a^   **Social Influence**   1. People close to me would recommend me to use an *online program*. ^a^ 2. My *community supervisor* would recommend me to use an *online program*. ^a^   **Facilitating Conditions**   1. I do have all necessary technical preconditions for using an *online program.* ^a^ 2. In case of technical problems with an *online program*, I would receive technical support. ^a^   **Internet Anxiety**   1. The internet is something threatening to me. ^a^ 2. I am afraid making an irrevocable mistake while using the internet. ^a^   **Attitude Towards Web-based Interventions**   1. Using the *online program* is a good idea. ^b^ 2. Using the *online program* would be interesting. ^b^ 3. Using the *online program* could be fun. ^b^ 4. I would like to work with the *online program*. ^b^   **Planning Behavior**   1. I have already made detailed plans when to complete the *sessions*. ^c^ 2. I have already made detailed plans how often I will work on the *sessions* during the week. ^c^ 3. I have already made detailed plans how to complete the *sessions* despite my other obligations and interests. ^c^ 4. I have already made detailed plans how to continue working on the *sessions* even if something comes up. ^c^   **Importance of Study Compensation**   1. Receiving money for @myTabu motivates me to participate in sessions. ^d^ 2. Receiving awards within @myTabu motivates me to participate in sessions. ^d^ |

*Note*. The adapted sections have been italicized. The questionnaire included a description of “online program”, stating that the term refers to a guided program that can be completed online and that, while in community supervision, helps individuals avoid recidivism and live crime-free lives. The program consists of individual sessions that are unlocked weekly.

^a^ Baumeister et al. (2014). ^b^ Apolinário-Hagen et al. (2019). ^c^ Zarski et al. (2018).  ^d^ self-constructed.
